# Presentation, management and outcomes of acute coronary syndrome: a registry study from Kenyatta National Hospital in Nairobi, Kenya

**DOI:** 10.5830/CVJA-2018-017

**Published:** 2018

**Authors:** Bahiru Ehete, D Huffman Mark, Temu Tecla, Gitura Bernard, Farquhar Carey, Bukachi Frederick

**Affiliations:** Northern Pacific Global Health Research Fellowship Training Consortium, University of Washington, Seattle, WA, and Division of Cardiology, Department of Medicine, David Geffen School of Medicine, University of California, Los Angeles, USA; Department of Preventive Medicine, Northwestern University, Chicago, IL, USA; Department of Global Health, University of Washington, Seattle, WA, USA; Kenyatta National Hospital, Division of Cardiology, Department of Medicine, Nairobi, Kenya; Departments of Global Health, Epidemiology and Medicine, University of Washington, Seattle, WA, USA; Department of Medical Physiology, University of Nairobi, Nairobi, Kenya

**Keywords:** acute coronary syndrome, sub-Saharan Africa, global health

## Abstract

**Background:**

Acute coronary syndrome (ACS) is understudied in sub-Saharan Africa despite its increasing disease burden. We sought to create an ACS registry at Kenyatta National Hospital to evaluate the presentation, management and outcomes of ACS patients.

**Methods:**

From November 2016 to April 2017, we conducted a retrospective review of ACS cases managed at Kenyatta National Hospital between 2013 and 2016, with a primary discharge diagnosis of ACS, based on International Classification of Diseases (ICD) 10 coding (I20-I24). We compared the presentation, management and outcomes by ACS subtype using analysis of variance testing. We created multivariable logistic regression models using the Global Registry of Acute Coronary Events (GRACE) risk score to evaluate the association between clinical variables, including guideline-directed medical therapy and in-hospital outcomes.

**Results:**

Among 196 ACS admissions, the majority (65%) was male, and the median age was 58 years. Most (57%) ACS admissions were for ST-segment-elevation myocardial infarction (STEMI). In-hospital dual antiplatelet ( > 85%), beta-blockade (72%) and anticoagulant (72%) therapy was common. A minority (33%) of patients with STEMI was eligible for reperfusion therapy but only 5% received reperfusion. In-hospital mortality rate was 17%, and highest among individuals presenting with STEMI (21%). After multivariable adjustment, higher serum creatinine level was associated with higher odds of in-hospital death (OR = 1.84, 95% CI: 1.21– 2.78), and STEMI and Killip class > 1 were associated with in-hospital composite of death, re-infarction, stroke, major bleeding or cardiac arrest (STEMI: OR = 8.70, 95% CI: 2.52–29.93; Killip > 1: OR = 10.7, 95% CI: 3.34–34.6).

**Conclusions:**

We describe the largest ACS registry at Kenyatta National Hospital to date and identify potential areas for improved ACS care related to diagnostics and management to optimise in-hospital outcomes.

Sub-Saharan Africa is increasingly facing a dual disease burden of infectious and non-communicable chronic diseases (NCDs), including ischaemic heart disease, which is the leading cause of deaths globally.^1^ The prevalence of ischaemic heart disease is steadily rising in sub-Saharan Africa due to the increasing prevalence of risk factors, including diabetes, obesity, smoking, physical inactivity, hypertension and dyslipidaemia in the context of urbanisation and globalisation.^1^ The prevalence and mortality rates of ischaemic heart disease in sub-Saharan Africa are predicted to rise by 70% in African men and 74% in African women by 2030.^2^

While the increasing burden of ischaemic heart disease in sub-Saharan Africa is recognised, few studies have evaluated the presentation, management and outcomes of acute manifestations of ischaemic heart disease, such as acute coronary syndrome (ACS). Accurate and timely assessment of ACS disease burden and current management trends in sub-Saharan Africa can help national and regional healthcare systems build capacity to respond appropriately to the rising epidemic of ischaemic heart disease in the region.^3^

Internationally, ACS registries have been valuable in studying the presentation, management and outcomes of patients for quality-improvement purposes.^3^ Data from large ACS registries in sub-Saharan Africa are limited, particularly public hospitals in Kenya. Societies such as the Pan-African Society of Cardiology (PASCAR) have recognised the need for improved understanding of ACS in the region and are advocating for initiatives including large-scale ACS registries.^4^

To improve current understanding of ACS management in Kenya, we sought to create an ACS registry at Kenyatta National Hospital, a major public referral centre, to evaluate the presentation, management and outcomes of patients with ACS.

## Methods

From November 2016 to April 2017 we conducted a retrospective chart review of ACS cases managed at Kenyatta National Hospital from 2013 to 2016. We used the existing electronic disease code database to identify ACS cases, using primary discharge codes (I20-I24) from the World Health Organisation International Classification of Diseases (ICD-10) system.^5^

The diagnosis of ACS subtype was made by the primary treating physician at the time of the index hospitalisation, based on the Third Universal Definition of Myocardial Infarction.^6^ We excluded cases that had a primary diagnosis of ACS, but upon further review of the medical admission, most likely had myocardial infarction secondary to a non-ACS aetiology (i.e. a non-type 1 myocardial infarction). In cases that the ACS subtype was not directly specified in the medical record, the primary data extractor (EB) reviewed each clinical presentation, biomarkers and ECG findings and determined the ACS subtype based on the Third Universal Definition of Myocardial Infarction.^6^

We included adults over 18 years with a diagnosis of ACS admitted and managed between 2013 and 2016. We abstracted demographics, presentation, self-reported medical history, diagnostics, treatment data and in-hospital clinical events. We used combined paper and electronic data-capture systems to abstract data from the medical record, which was performed by one author (EB).

We defined guideline-directed in-hospital medical therapy as receiving a combination of aspirin, a second antiplatelet (e.g. clopidogrel, ticagrelor or prasugrel), beta-blocker within 24 hours of presentation and anticoagulation at any point during the hospitalisation. We defined guideline-directed discharge medical therapy as receiving a combination of aspirin, second antiplatelet drug, beta-blocker and statin. We assessed in-hospital outcomes, including in-hospital death and in-hospital major adverse cardiovascular events (MACE), defined as the composite of in-hospital death, re-infarction, stroke, heart failure, major bleeding or cardiac arrest.

We acquired ethics approval to conduct this research from the Kenyatta National Hospital and University of Nairobi ethics research committee (KNH-UON ERC), Northwestern University institutional review board, and University of Washington institutional review board. Informed consent was waived based on the retrospective nature of the study for the collection of anonymised data.

## Statistical analysis

We present continuous data as means (standard deviation) or median (range or interquartile range) when skewed, and categorical data as proportions. Comparisons by ACS subtype were made via analysis of variance for continuous variables and chi-squared testing for categorical variables. We created multivariable logistic regression models using the Global Registry of Acute Coronary Events (GRACE) risk score to evaluate the association between clinical variables, including guidelinedirected medical therapy and in-hospital death or major adverse cardiovascular events.^4^ We defined statistical significance using a two-sided p-value < 0.05. We used Stata version 14.0 (StataCorp, LLC. College Station, TX).^5^

## Results

**Fig. 1:**
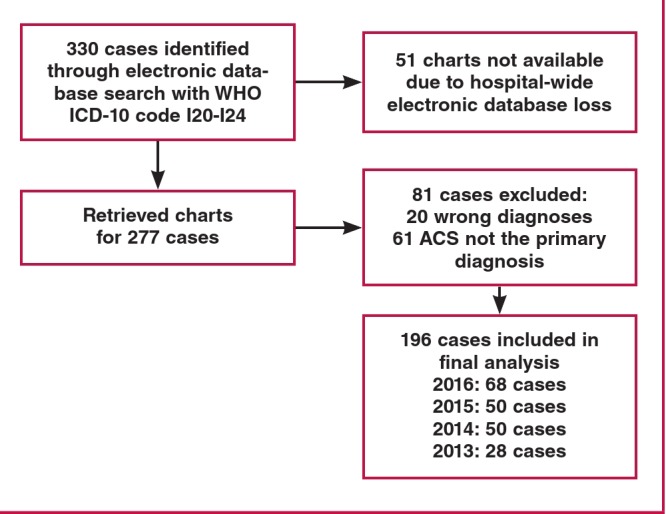
Study flow chart of ACS cases admitted to Kenyatta National Hospital between 2013 and 2016.

Fig. 1 demonstrates the flow of participants in this study. We identified a total of 330 admissions that met our study criteria. We could only retrieve partial admission data from 2013 due to a hospital-wide electronic database loss that occurred between 2011 and 2013, which led to the exclusion of 51 cases. A further 81 cases were excluded because of incorrect diagnosis (20) or ACS not being the primary discharge diagnosis (61). We therefore included 196 cases in our final analysis.

Table 1 summarises patients’ baseline characteristics by ACS subtype. The majority (57%) of the cases were ST-elevation myocardial infarction (STEMI) followed by non-ST-elevation myocardial infarction (NSTEMI, 26%) and unstable angina (UA, 12%). Cases without an ECG but with positive biomarkers and clinical presentation consistent with ACS represented 5% of cases. Most participants (64%) were men, and the median age (IQR) was 58 (48–68) years. More than one-third (38%) of all admissions were transferred from an outside hospital.

**Table 1:** Basic characteristics of patients admitted with ACS at Kenyatta National Hospital between 2013 and 2016 by ACS subtype

*Variables*	*All n = 196*	*STEMI n = 112*	*NSTEMI n = 50*	*UA n = 24*	*Biomarker (+) only n = 10*	*p-value*
Type of ACS	196	112 (57)	50 (26)	24 (12)	10 (5)	
Age, years (median, IQR)	57.5 (48, 68)	60 (53, 69)	56.5 (44, 68)	51.5 (48, 67)	62.5 (45, 65)	0.18
Male, n (%)	127 (65)	81 (65)	31 (24)	9 (8)	6 (5)	0.01
Transferred, n (%)	74 (38)	56 (50)	11 (22)	5 (21)	2 (20)	< 0.001
History of hypertension, n (%)	124 (63)	67 (60)	34 (680	18 (75)	5 (50)	0.34
History of diabetes, n (%)	80 (41)	53 (47)	16 (32)	9 (38)	2 (20)	0.14
History of stroke, n (%)	1 (0.5)	1 (0.9)	0	0	0	0.86
History of end-stage renal disease, n (%)	4 (2)	0	2 (4)	2 (8)	0	0.03
History of smoking, n (%)	17 (9)	13 (12)	3 (6)	1 (4)	0	0.07
Heart rate, bpm (median, IQR)	84 (72–101)	84 (72–103)	86 (100–73)	90 (95–76)	79 (66–101)	0.79
Systolic blood pressure, mmHg (median, IQR)	137 (116–156)	136 (114–155)	143 (116–156)	138 (103–156)	150 (132–172)	0.41
Killip class > 1, n (%)	73 (39)	50 (44)	17(34)	5 (21)	5 (50)	0.07

STEMI: ST-elevation myocardial infarction, NSTEMI: non-ST-elevation myocardial infarction, UA: unstable angina, bpm: beats per minute.

Hypertension (63%) was the most common co-morbidity, followed by diabetes (41%). Smoking rates were low across all groups (9%); however, 27% had undocumented smoking status, which likely led to underestimation of the overall smoking prevalence. The proportion of patients who presented in heart failure with Killip class > 1 was highest among STEMI cases (44%).

Table 2 shows a summary of the in-hospital management of ACS patients. We stratified the data into four groups: key investigations, in-hospital acute medical therapy focusing on administration of guideline-directed medications within the first 24 hours of admission, in-hospital reperfusion therapy, and discharge medical therapy.

**Table 2:** In-hospital and discharge diagnostics, medical and reperfusion therapy, and rates of guideline-directed in-hospital and discharge medical therapy of ACS patients admitted to Kenyatta National Hospital between 2013 and 2016

*Variables*	*All n = 196*	*STEMI n = 112*	*NSTEMI n = 50*	*UA n = 24*	*Biomarker (+) only n = 10*	*p-value*
Key investigations						
ECG < 24 h, n (%)	152 (78)	95 (85)	37 (74)	20 (83)	–	< 0.001
Non-transferred	87 (71)	43 (77)	29 (74)	15 (79)	–	
Transferred	65 (84)	52 (93)	8 (73)	5 (100)	–	
Cardiac enzyme (+) in 24 h, n (%)	134 (68)	75 (67)	49 (98)	–	9 (90)	< 0.001
Echocardiography, n (%)	101(52)	61 (54)	28 (56)	10 (42)	2 (20)	0.13
LVEF < 40%, n (%)	33 (33)	25 (41)	6 (21)	2 (20)	0 (0)	< 0.001
In-hospital medical therapy						
Aspirin, n (%)	185 (94)	104 (93)	50 (100)	22 (92)	–	0.21
Second antiplatelet, n (%)	172 (88)	99 (88)	46 (92)	20 (83)	–	0.20
Beta-blocker, n (%)	137 (72)	79 (75)	32 (65)	18 (75)	–	0.68
Anticoagulation	140 (72)	85 (76)	38 (76)	15 (65)	–	< 0.001
Guideline-directed in-hospital medical therapy*, n (%)	58 (56)	34 (60)	22 (56)	10 (53)	2 (25)	
In-hospital reperfusion therapy						
Eligible for reperfusion, n (%)	–	37 (33)	–	–	–	
Thrombolysis, n (%)	–	2 (5)	–	–	–	
Diagnostic catheterisation, n (%)	17 (9)	12 (11)	4 (8)	1 (10)	0 (0)	
PCI, n (%)	2 (12)	0 (0)	1 (25)	1 (100)	–	
CABG, n (%)	0 (0)	0 (0)	0 (0)	0 (0)	0 (0)	
Medications on discharge						
Aspirin, n (%)	152 (96)	86 (99)	41 (91)	19 (86)	6 (100)	0.62
Second antiplatelet, n (%)	131 (82)	79 (91)	34 (76)	13 (59)	5 (83)	0.07
Beta-blocker, n (%)	115 (72)	67 (77)	25 (56)	17 (77)	6 (100)	0.04
Statin, n (%)	137 (86)	78 (90)	34 (76)	18 (82)	4 (67)	0.31
ACEI/ARB for LVEF < 40%, n (%)	19 (63)	13 (59)	5 (83)	1 (50)	–	0.41
Guideline directed discharge medical therapy**, n (%)	89 (56)	34 (64)	22 (47)	10 (41)	3 (60)	

ACS: acute coronary syndrome, STEMI: ST-elevation myocardial infarction, NSTEMI: non-ST-elevation myocardial infarction, UA: unstable angina, Biomarker (+ only: these are cases that presented with symptoms of ACS and had a positive biomarker test, however did not get an ECG during their hospitalisation, LVEF: left ventricular ejection fraction, PCI: percutaneous coronary intervention, CABG: coronary artery bypass graft, ACEI: ACE inhibitor, ARB: angiotensin receptor blocker.

*Guideline-directed in-hospital medical therapy includes patients who received aspirin, a second antiplatelet and a beta-blocker within 24 hours of presentation and an anticoagulant at any point during hospitalisation.

**Guideline-directed discharge medical therapy includes patients who received aspirin, a second antiplatelet, a beta-blocker and a statin at discharge.

Overall, 82% of all cases received an ECG within 24 hours of presentation, with higher rates among patients who were transferred versus non-transfer patients (88 vs 71%, p < 0.001). The proportion of patients that received an ECG within 24 hours of admission each year between 2013 and 2016 was 82, 80, 80 and 72%, respectively (p = 0.69). Cardiac biomarkers were measured in 86% of cases, and approximately half (52%) received echocardiography during their hospitalisation. A total of 10 cases were primarily diagnosed by symptoms and positive biomarkers without an ECG, with six of these cases managed in 2016, three in 2015, one in 2014 and none in 2013.

During the acute management phase, dual antiplatelet use was 87%. The rates of beta-blocker use (72%) within the first 24 hours of admission and anticoagulant use (72%; 80% enoxaparin) during hospitalisation were also relatively high. After excluding transfer patients, the rate of guideline-directed in-hospital medical therapy, defined as receiving aspirin, a second antiplatelet, beta-blocker within 24 hours of admission and an anticoagulant at some point during the hospitalisation was 56%.

A minority of overall (17 cases, 9%) and STEMI cases (12 cases, 11%) underwent in-hospital diagnostic cardiac catheterisation with only 12% undergoing percutaneous coronary intervention (one NSTEMI, one unstable angina). Using the 2013 American College of Cardiology/American Heart Association guidelines for the management of STEMI,^7^ we idenfied 37 (33%) STEMI cases eligible for reperfusion, half of whom were transfers. Two eligible STEMI cases (5%) received thrombolytic therapy and both were transferred from outside hospitals.

We assessed discharge medical therapy, focusing on guidelinedirected prescription of medications upon discharge and excluding patients who left against medical advice (n = 4). Discharge aspirin use was 96%, and second antiplatelet agent discharge use was 82%; combined dual antiplatelet use was 81%. Beta-blocker discharge use was 70%, and statin discharge use was 86%. The rate of guideline-directed discharge medical therapy defined as receiving aspirin, a second antiplatelet and beta-blocker therapy was also 56%. Among individuals with an ejection fraction less than 40% (n = 33), 63% received an ACE inhibitor or angiotensin receptor blocker (ARB). Among patients with ejection fraction less than 40%, the rate of guideline-directed medical therapy with simultaneous dual antiplatelet, beta-blocker, statin and ACE inhibitor or ARB use was 48%.

The overall in-hospital mortality rate was 17%, with a gradient in mortality rate by ACS subtype (STEMI 21%, NSTEMI 10%, UA 9%, biomarker positive only 30%, p = 0.16) (Table 3). The rate of MACE, defined as death, re-infarction, stroke, cardiogenic shock, major bleeding or cardiac arrest was 40%, with a similar gradient by ACS subtype (STEMI 54%, NSTEMI 20%, UA 7%, biomarker positive only 30%, p < 0.001) (Table 3).

**Table 3:** In-hospital mortality and major adverse cardiovascular events and association between in-hospital guideline-directed therapy and in-hospital outcomes of ACS patients admitted and managed at Kenyatta National Hospital between 2013 and 2016

	*In-hospital mortality*	*In-hospital MACE*
All, n (%)	33 (17)	78 (40)
STEMI, n (%)	23 (21)	61 (54)
NSTEMI, n (%)	5 (10)	11 (22)
UA, n (%)	2 (8)	3 (13)
BM (+) only	3 (30)	3 (30)
*Guideline-directed in-hospital medical therapy, n (%)	8 (12)	27 (40)
Non-guideline-directed in-hospital medical therapy, n (%)	8 (15)	14 (26)
Guideline-directed vs non-guideline-directed, OR (95% CI)	0.76 (0.27–2.20)	1.88 (0.86–4.10)

ACS: acute coronary syndrome, STEMI: ST-elevation myocardial infarction, NSTEMI: non-ST-elevation myocardial infarction, UA: unstable angina, BM (+): biomarker positive only: these are cases that presented with symptoms of ACS and had a positive biomarker test, however did not get an ECG during their hospitalisation, MACE: major adverse cardiovascular events.

*Guideline-directed in-hospital medical therapy includes patients who received aspirin, a second antiplatelet and a beta-blocker within 24 hours of presentation and an anticoagulant at any point during hospitalisation.

Table 4 summarises variables assessed as potential predictors of in-hospital mortality before and after adjustment using the GRACE risk score. After multivariable adjustment, higher serum creatinine level was associated with higher odds of in-hospital death (OR = 1.84, 95% CI: 1.21–2.78), and Killip class > 1 was associated with in-hospital composite of death, re-infarction, stroke, major bleeding or cardiac arrest (STEMI: OR = 4.71, 95% CI: 2.46–9.02; Killip > 1: OR = 10.7, 95% CI: 3.34–34.6).

**Table 4:** Predictors of in-hospital death and major adverse cardiovascular events (MACE) including death, re-infarction, stroke, heart failure, cardiogenic shock, major bleeding and cardiac arrest of ACS patients admitted to Kenyatta National Hospital between 2013 and 2016

	*Unadjusted OR (95% CI)*		*Adjusted (for age, gender and GRACE risk score variables) OR (95% CI)*	
Variables	In-hospital death, n = 33	In-hospital MACE, n = 78	In-hospital death, n = 33	In-hospital MACE, n = 78
Age (per year)	1.03 (1.0–1.06)*	1.00 (1.01–1.05)*	1.04 (0.98–1.11)	1.01 (0.98–1.06)
Heart rate (per bpm)	0.98 (0.97–1.00)	1.00 (0.99–1.02)	1.02 (0.99–1.11)	1.01 (098–1.04)
SBP (per mmHg)	0.99 (0.98–1.00)	0.99 (0.98–1.00)	0.96 (0.93–0.98)	0.98 (0.96–0.99)
Serum Cr (per mg/dl)	1.35 (1.12–1.66)*	1.13 (0.94–1.34)	1.84 (1.21–2.78)*	1.04 (0.84–1.31)
Killip class 1 vs > 1	5.80 (2.5–13.7)*	11.45 (0.80–22.4)*	1.8 (0.42–8.14)	10.7 (3.34–34.6)*
Positive cardiac enzyme	2.60 (0.58–11.8)	2.23 (0.89–5.63)	0.91 (0.94–8.80)	1.42 (0.35–5.73)
ST-segment deviation	2.11 (0.85–5.22)	5.35 (2.60–10.99)*	3.12 (0.57–16.87)	1.72 (0.12–24.40)
STEMI vs UA (ref)	2.84 (062–2.98)	8.37 (2.36–29.70)*	0.77(0.02–38.56)	1.71 (0.21–13.80)

*p-value < 0.05; GRACE risk score variables: age, gender, SBP, HR, positive cardiac enzyme, ST-segment change, creatinine, cardiac arrest.

SBP: systolic blood pressure, HR: heart rate, Cr: creatinine, STEMI: ST-segment myocardial infarction.

We also evaluated the association between receiving guidelinedirected in-hospital medical therapy and in-hospital MACE level using logistic regression and adjusting for covariates in the GRACE risk score. We did not demonstrate an association between in-hospital death and combined in-hospital death and MACE before and after multivariable adjustment (OR = 0.77, 95% CI: 0.27–2.20; OR = 1.88, 95% CI: 0.86–4.10, respectively), but these results were imprecise and were likely driven by the small sample size and number of events.

## Discussion

Through this retrospective chart review we report the presentation, management and outcomes of ACS patients managed at Kenyatta National Hospital between 2013 and 2016. Most patients were men in their late 50s presenting with STEMI. Approximately one out of every five patients did not receive an ECG within the first 24 hours, and one out of every 20 patients did not receive an ECG at all. While more than one out of every two patients received echocardiography, the gap in ECG care represents an opportunity for diagnostic improvement. Rates of in-hospital medical therapy were relatively high but reperfusion rates among eligible individuals were low. Increasing timely, appropriate reperfusion therapy for eligible STEMI patients may be an important area of focus because of the high mortality rate demonstrated among these patients.

The median age of presentation found in this study is similar to other studies from sub-Saharan Africa, which demonstrate that ACS cases in sub-Saharan Africa tend to present at a younger age, typically in their 50s, compared to high-income countries, which have a median age in the mid-to-late 60s.^4^ A 2010 retrospective study by Ogeng’o et al. at Kenyatta National Hospital of 120 ACS cases admitted between 2000 and 2009 reported the mode of diagnosis, demographics, risk factors and in-hospital heart failure and mortality rates.8 The mean age in this study was 56.8 years with a similar 2:1 male-to-female ratio.

Our study also demonstrates a doubling in rates of hypertension and diabetes (63 and 41%, respectively) compared to the 35 and 21% rates reported in the Ogeng’o study, while smoking rate was similarly low (9 and 13%, respectively).^8^ The 2010 Ogeng’o study did not specify ACS subtypes and overall rates of in-hospital diagnostics such as ECG, echocardiography and coronary angiography, and therefore we were unable to make comparisons in those areas. However, total mortality rate demonstrated in our study was notably higher (17%) compared with the previous report of 5%.^8^

The 2004 INTERHEART study, a multi-continental case–control study, which incorporated nine countries from sub-Saharan Africa, including Kenya, demonstrated that acute myocardial infarction risk factors among the sub-Saharan African cohort were similar to that of the overall study population. However, a history of hypertension was associated with increased myocardial infarction risk among the black African group compared to the general study population.^9^

The high frequency of STEMI (57%) presentation demonstrated in this study is also similar to other studies from sub-Saharan Africa, including a 2012 prospective study of 111 ACS admissions from the Aga Khan University Hospital, a private institution in Nairobi (56%, n = 111).^10^ The Acute Coronary Events – a Multinational Survey of Current Management Strategies (ACCESS) registry is another largescale multi-national study that included 642 patients from South Africa. This study had 41% STEMI, 32% NSTEMI and 27% unstable angina cases.

Rates of in-hospital medical therapy such as aspirin and beta-blocker use demonstrated in this study are comparable to the ACCESS-South Africa cohort findings; however, there are important differences in reperfusion rates. The Aga Khan University study demonstrated a 68% reperfusion rate [either percutaneous coronary intervention (PCI) or thrombolysis], while the ACCESS registry reported 96% in-hospital reperfusion rate with thrombolysis and/or PCI.^11^ Caution must be applied when comparing these reperfusion rates to our study, given the likely significant patient- and hospital-level socio-economic variation across these studies.

Future directions of study include evaluating initiatives for quality improvement related to diagnostics (e.g. ECG evaluation of all patients with chest pain) and management (e.g. reperfusion of eligible patients). One integral component includes an ongoing, prospective ACS registry to assess time trends in presentation, management and outcomes and devise future quality-improvement initiatives.

Internationally, results of large-scale registries such as GRACE-ACS^4^ have contributed significantly to better understanding of ACS presentation, management and outcomes and have led to the design of other ACS registries globally, including in low-middle-income countries such as the Kerala ACS registry,^13^ China Acute Myocardial Infarction (CAMI) registry^14^ and the Registry for Acute Coronary Syndrome Events in Nigeria (RACE-Nigeria) from a sub-Saharan African country.^2^ Cardiology societies in sub-Saharan Africa including the Kenyan Cardiac Society (KCS) and the Pan-African Society of Cardiology (PASCAR) have recognised the need for data on ACS and are advocating for initiatives to build local and regional ACS registries to have improved understanding of disease presentation, management and outcomes in the region.

ACS registries in both high- and low-middle-income countries have also led to subsequent quality-improvement initiatives. These include ACS quality-improvement randomised, control trials, such as the Brazilian Intervention to Increase Evidence Usage in Acute Coronary Syndromes (BRIDGE-ACS),^15^ the Clinical Pathways for Acute Coronary Syndromes, phases 2 and 3 (CPACS-2 and -3)^16^ in China, and the Acute Coronary Syndrome Quality Improvement in Kerala (ACS QUIK) study in India.^17^

These multi-institutional randomised, control trials have investigated the impact of quality-improvement tools such as clinical pathways, audits and performance feedback on both processes of care and outcomes, with the goal of improving ACS management. Such future efforts within sub-Saharan Africa could be instrumental in identifying unique solutions tailored to the needs and capacity of the region to improve ACS care. Through this research process, we have engaged with key stakeholders at Kenyatta National Hospital within the division of cardiology and department of research to assess the existing research infrastructure and capacity, to improve processes and outcome measures of patients with ACS.

## Strengths and limitations

This study, the largest study at Kenyatta National Hospital to date, has assessed the presentation, management and outcomes of ACS patients managed at the hospital. The main study limitation is based upon the retrospective design of the study. Like most hospitals in the region, Kenyatta National Hospital uses paper charts for medical records, and we were not able to locate 51 charts that met our study criteria. Additionally, there was loss of electronic disease code database at Kenyatta National Hospital in 2013, which resulted in only 40 admissions being identified from 2013. However, it is unlikely that these omissions would have influenced the overall findings from this study. One author (EB) made assessments to include and exclude cases and completed the data extraction, which adds another potential limitation to data quality.

## Conclusions

This is the largest study at Kenyatta National Hospital to evaluate the presentation, management and outcomes of ACS patients managed at a public referral hospital that provides care to a diverse pool of patients in Kenya. The findings present opportunities for future quality-improvement initiatives, especially in the areas of initial diagnostic capabilities and reperfusion therapy. A prospective ACS registry and linked quality-improvement programme would be valuable to improve quality and safety of ACS patients and as a model for other cardiovascular conditions.
